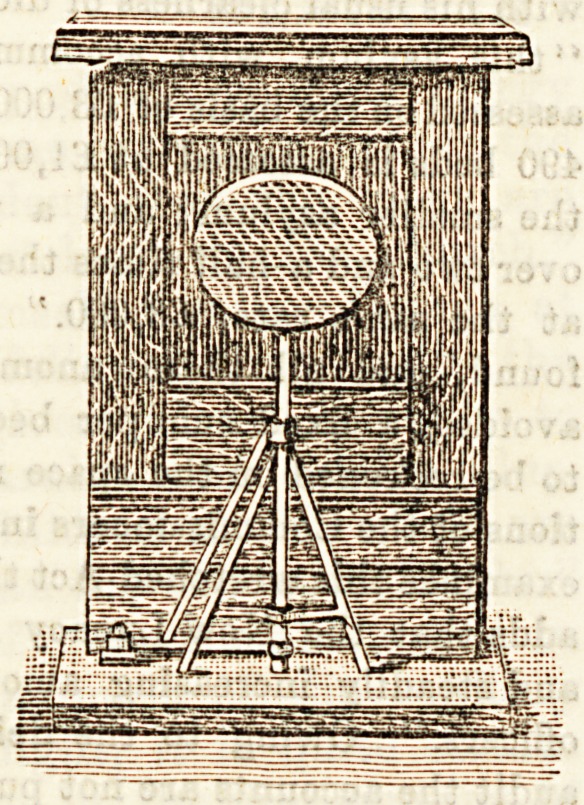# New Drugs, Appliances, and Things Medical

**Published:** 1892-02-06

**Authors:** 


### NEW DRUGS, APPLIANCES, AND THINGS MEDICAL.

### THE "HUMANE" SEAT.

Can a seat, strictly speaking, be "humane"? The severe metaphysician would say decidedly not. Nevertheless, even a seat may be so necessary, so appropriate, and so beneficial that one may properly call its inventor, at any rate, and perhaps even itself "humane." Is there any grown-up person living who has not sometimes pitied shop assistants? To stand behind the counter for ten or twelve hours a day, and for six days a-week, with no opportunity of sitting down, except for the briefest of meals, is for all but the strongest of the strong little short of martyrdom. The world is a stern place for some people, and not the least stern for shop assistants. But we have the good fortune to live in an age of kindly and progressive humanity. The daily papers have long cried out for the young men and women of our shops: and a sympathising and considerate public has almost universally echoed the cry. In response to this general demand a "humane seat" has at last been produced, and we present a sketch of it herewith. An imperative condition of the general adoption of the seat is that it must be perfectly convenient and efficient. If it be so constructed as that it "gets in the way," or that its frequent use causes a considerable loss of time we may be quite certain that the stern necessities of competitive business will refuse to sanction its adoption. The "humane" seat seems to us to excellently fulfil the conditions here laid down. It is attached to the back portion of the counter; it is not a moveable shelf, but a stool with legs; it can be brought out for use in a moment by a mere turn of the hand; and it can be replaced in its position, out of sight and out of the way by another turn of the hand. We have carefully inspected the stool, and the inventor has demonstrated to us both its efficiency as a seat and the convenient rapidity with which it can be brought into use. We very cordially commend an experimental consideration of it to all those who employ shop assistants.

### SWINBORNE'S ISINGLASS.

During the present epidemic one is frequently asked for some variation in the food ordered for patients. They weary of milk, of beef-tea and peptonized foods, and are glad to get some change. This they will find in the above. We have recently examined both the isinglass and calves' feet gelatine, and find them perfectly pure and admirably adapted for the purpose of making good jelly. A large proportion of influenza patients have some form or the other of catarrhal throat condition. Well-made jellies relieve them even more than medicines, and we cannot say anything stronger than that Swinborne's preparations are admirably adapted for this purpose. Nothing is nicer than a mouthful of clear cool jelly, it seems often to be just the thing when nothing else can be taken, and, of course, its nutritive power is great; though other forms of primary nourishment must be given, gelatine (and isinglass is only the finest variety of gelatine, being fish derived instead of animal) will carry on the work until the stomach is in a position to tolerate stronger foods. A jelly has another advantage, it can be combined with other forms of nutriment and the flavour changed, so that the patient can be tempted in a number of ways to feed himself up, a great difficulty in the present outbreak of "the acute contagious fever" called influenza.

## Figures and Tables

**Figure f1:**
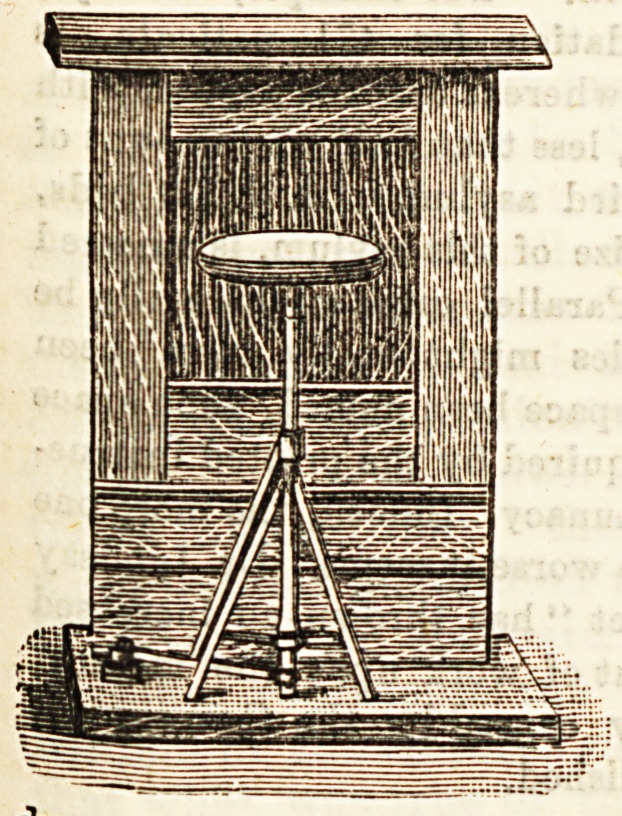


**Figure f2:**